# 1761. A Pre-Post Interventional Study on the Impact of Asynchronous Microlearning of Antimicrobial Stewardship Principles among Nursing Staff at a Large Academic Medical Center

**DOI:** 10.1093/ofid/ofac492.1391

**Published:** 2022-12-15

**Authors:** Laura Bobbitt, Christo Cimino, Kim Garvey, Leanna Craft, Nicole Eichenseer, George E Nelson

**Affiliations:** Vanderbilt University Medical Center, Nashville, Tennessee; Vanderbilt University Medical Center, Nashville, Tennessee; Vanderbilt University Medical Center, Nashville, Tennessee; Vanderbilt University Medical Center, Nashville, Tennessee; Vanderbilt University Medical Center, Nashville, Tennessee; Vanderbilt University Medical Center, Nashville, Tennessee

## Abstract

**Background:**

Nurses perform several functions that are integral for antimicrobial stewardship (AS). However, nurses are underrepresented in research and underutilized in AS interventions and implementation. Additionally, lack of education and training is consistently cited as a barrier to full nurse participation in AS activities. We used a novel approach for nursing AS education by utilizing an asynchronous mobile learning platform which delivered small amounts of information to participants daily via e-mail or text message. The objective of this study was to determine the effect of this innovated AS microlearning pedagogical approach on nursing staff knowledge, attitudes, and practices (KAP) regarding AS principles.

**Methods:**

A course consisting of 9 case-based multiple-choice questions was developed. One question per day was delivered to participants via an institutional web-based application and followed by a brief educational explanation on associated AS principles. A KAP survey with 20 Likert scale questions on a 5-point scale was administered to participants pre-and post-intervention. Survey results were compared using a Wilcoxon signed-rank test.

**Results:**

Of the 46 participants, most (86%) reported they administer antibiotics to >50% of their patients. Of the 20 KAP questions, participant’s mean score post-intervention demonstrated statistically significant improvement for 90% (18/20) of the items. Participant’s confidence improved in key AS activities including differentiating between colonization and infection (P< 0.001), identifying unnecessary urine cultures and inappropriate treatment of urinary tract infections (P< 0.001), recognizing opportunities for intravenous to oral therapy conversion (P< 0.001), and assessing for antibiotic-associated adverse effects (P< 0.001).

**Conclusion:**

Inclusion of nurses in AS is critical. AS nursing education through an asynchronous, brief educational format via a mobile platform resulted in statistically significant improvement in most topics covered. After course completion, participants felt more confident in their ability to participate in key AS activities. This study forms the basis for expanded AS educational efforts for all healthcare professionals.

**Disclosures:**

**All Authors**: No reported disclosures.

